# Factors associated with suboptimal adherence to antiretroviral therapy in Viet Nam: a cross-sectional study using audio computer-assisted self-interview (ACASI)

**DOI:** 10.1186/1471-2334-13-154

**Published:** 2013-03-27

**Authors:** Hoa M Do, Michael P Dunne, Masaya Kato, Cuong V Pham, Kinh V Nguyen

**Affiliations:** 1 Hanoi School of Public Health, Hanoi, Viet Nam; 2 Queensland University of Technology, School of Public Health, Brisbane, Australia; 3 World Health Organization Representative Office in Viet Nam, Hanoi, Viet Nam; 4 National Hospital for Tropical Diseases, Hanoi, Viet Nam

**Keywords:** ACASI, Adherence, Antiretroviral therapy, Depression, Substance use, Viet Nam

## Abstract

**Background:**

Optimal adherence to antiretroviral therapy (ART) is necessary for people living with HIV/AIDS (PLHIV). There have been relatively few systematic analyses of factors that promote or inhibit adherence to antiretroviral therapy among PLHIV in Asia. This study assessed ART adherence and examined factors associated with suboptimal adherence in northern Viet Nam.

**Methods:**

Data from 615 PLHIV on ART in two urban and three rural outpatient clinics were collected by medical record extraction and from patient interviews using audio computer-assisted self-interview (ACASI).

**Results:**

The prevalence of suboptimal adherence was estimated to be 24.9% via a visual analogue scale (VAS) of past-month dose-missing and 29.1% using a modified Adult AIDS Clinical Trial Group scale for on-time dose-taking in the past 4 days. Factors significantly associated with the more conservative VAS score were: depression (p < 0.001), side-effect experiences (p < 0.001), heavy alcohol use (p = 0.001), chance health locus of control (p = 0.003), low perceived quality of information from care providers (p = 0.04) and low social connectedness (p = 0.03). Illicit drug use alone was not significantly associated with suboptimal adherence, but interacted with heavy alcohol use to reduce adherence (p < 0.001).

**Conclusions:**

This is the largest survey of ART adherence yet reported from Asia and the first in a developing country to use the ACASI method in this context. The evidence strongly indicates that ART services in Viet Nam should include screening and treatment for depression, linkage with alcohol and/or drug dependence treatment, and counselling to address the belief that chance or luck determines health outcomes.

## Background

Most studies examining the prevalence and determinants of antiretroviral therapy (ART) adherence have been conducted in the United States, Europe and Africa, but relatively few have been performed in Asia [1]. We have identified only 16 published articles on ART adherence in Asia from 2002–2011. In general, the estimates from Asia indicate that ART adherence is substantially higher than adherence in the United States and Europe, and somewhat higher than adherence in Africa. In Asia, these studies reported that suboptimal adherence in the week or month preceding interview was 0.4%–26%, with most studies reporting suboptimal adherence under 15%. High adherence estimates in Asia may arise from adherence over-reporting due to the common practice of face-to-face interviewing in clinical environments, often where health care providers are present or even conduct interviews [2,3]. Recent studies in Viet Nam have highlighted these problems and drawn attention to the need for a more confidential approach to data collection [4].

In Viet Nam, the number of PLHIV receiving ART rapidly expanded from 500 in 2004 to more than 49,000 in early 2011. To date, only two published studies in Viet Nam have estimated the prevalence of ART suboptimal adherence. Jordan et al. recruited 100 current or former injection drug users in Hanoi and found that 85% were optimal adherers [5]. The second study followed a cohort of 248 PLHIV at two out-patient clinics in Ho Chi Minh City and reported an adherence of over 98% [4]. Neither study in Viet Nam quantitatively examined determinants of ART adherence.

In their systematic review, Simoni et al. recommended that information on ART adherence be collected using a method like the audio computer-assisted self-interview (ACASI) [6]. ACASI is a computerized method for administering questionnaires. It has been widely used in health studies in both developed and developing countries, especially on sensitive topics such as HIV/AIDS, other sexually transmitted diseases, and drug use [7-9]. Instead of providing answers directly to interviewers, the respondents simultaneously read and listen to questions via headphones and type their answers, without being witnessed or overheard by others [7]. ACASI maximizes patients’ anonymity, minimizes the influence of health care providers in the data collection process and eases patients’ concerns about the impact of the interview on their treatment [6,9]. Thus, ACASI reduces social desirability response bias compared with in-person interviews [8,10]. In Viet Nam, this technique has been shown to be acceptable in studies of sensitive health issues with both adolescents and injection drug users [9,11].

In this study we assess the level of suboptimal adherence to ART among PLHIV and examine the influence of a range of possible determinants of ART adherence in Northern Viet Nam, using the ACASI technique.

## Methods

### Study sites

The study was carried out in five outpatient clinics in two provinces in northern Viet Nam. The sites included Dong Da Hospital and Dong Anh District Health Center in Hanoi City (urban area) and Hai Duong Provincial AIDS Center, Kim Mon District Health Center and Chi Linh District Health Center in Hai Duong Province (rural areas). These five clinics provided ART services to 1005 HIV/AIDS patients. The two clinics in Hanoi received support from the President’s Emergency Plan for AIDS Relief, and the three clinics in Hai Duong received support from the Global Fund for AIDS, Tuberculosis and Malaria.

### Study participants

Patients were eligible for the study if they were aged 18 years or older and they had received ART for at least 3 months. Patients were excluded if they had any serious health problems that impaired their ability to answer the questionnaire by themselves, were unable to come to the study sites, or refused to participate. The study used a clinic-based cross-sectional design. The sample size was calculated using the WHO Sample Size Determination for Health Studies formula [12]:

$$n=\frac{{Ζ}_{1-\alpha /2}^{2}p\left(1-p\right)}{d^{2}}$$

The following parameters were used to calculate the sample size: proportion of adherence (P) among HIV/AIDS patients in Viet Nam, which has been estimated to be about 85% [13], absolute precision (d) of 5% and a confidence level (z^2^_ 1-_α_/2)_ of 95%. This gave a sample size of 289. To increase the statistical power and to incorporate a design effect due to clustering within clinics, the sample size was multiplied by two (N = 578). In anticipation of incomplete interviews we then increased the final sample size by 5% (29 cases), yielding a minimum survey sample of 607 people. The number of patients studied per clinic was proportional to the number of eligible patients in each clinic. Eligible patients were contacted and invited to participate in the study when they came to the out-patient clinics to pick up their monthly medications. Patients were recruited consecutively between December 2008 and March 2009 until the minimum sample size was reached. This study was approved by the Research Ethics Committees of the Hanoi School of Public Health in Viet Nam and the Queensland University of Technology in Australia.

### Data collection

The survey instruments were field tested, and validated, as previously described [14]. All interview questions related to ART adherence and potential determinants of ART adherence were voice-recorded in the ACASI program developed specifically for this study using Visual Basic and Microsoft Access. A simple set of equipment, comprising a computer with installed ACASI software, a mouse, and headphones, was provided to all respondents. Participants listened to questions via headphones while simultaneously reading them on a computer monitor, and entered responses directly into the computer without verbalizing their answers.

The ACASI interviews were conducted in private rooms, and respondents were told that their information would be kept confidential. Data collection was performed by researchers who were not involved in the provision of care, and no health care providers were involved in the interviews or handling the data. To complement data from the ACASI interviews, some clinical information was extracted through review of medical records, with patients’ informed consent. The ACASI technique proved to be highly acceptable to patients, and most participants required only a few minutes to familiarize themselves with the method. This technique also eliminated interviewer variation that could occur when multiple researchers conduct face to face interviews, and also prevented potential data entry errors.

### ART adherence measurement

The study used two measures of ART adherence through self-report. First, a visual analogue scale (VAS) was adopted from Walsh et al. [15]. Respondents were asked to specify the percentage of ART doses taken in the past month by indicating a position along a continuous line between 0% and 100%.

Second, the Adult AIDS Clinical Trials Group (AACTG) adherence baseline questionnaire was adapted with minor modification, as a means to cross-validate the VAS adherence estimate [16]. While the original AACTG questionnaire asks for the number of missing doses in the past 4 days, in the present study respondents were asked about the number of doses they did not take on time in the past 4 days. The level of adherence was defined as the proportion of prescribed pills actually taken on time (i.e., within 1 hour of scheduled dose time) over a 4-day period. We used this time range because a previous study found that the proportion of prescribed doses taken on time (i.e., [number of doses taken ±1 h of dose time]/[total number of prescribed doses]) was found to be strongly associated with viral suppression [17].

Both the VAS and the AACTG questionnaire were selected for this study because they are the most commonly used tools in ART adherence studies worldwide [6]. Both are valid in a variety of clinical settings because they lead to similar estimates of the relationship between adherence and viral suppression [18-20]. An adherence of less than 95% was classified as non-adherent in this study. This threshold was set because adherence of less than 95% is associated with virologic failure and thus considered sub-optimal [21].

### Measurements of predictor variables

Variables potentially associated with ART adherence were selected based on a global literature review [1] and findings from a qualitative study previously conducted in Viet Nam [14]. The first group of variables were individual factors: gender, age, education, occupation, marital status, alcohol use, illicit drug use, depression, chance health locus of control (i.e., the degree to which individuals believe that their health status is controlled by chance or luck), and use of adherence reminder tools (e.g., clock or cell phone alarm, cell phone message, television or radio program). The second group of variables were clinical and health system factors: ART regimen, duration on therapy, medication side-effect experiences, current tuberculosis (TB) treatment, cotrimoxazole use, clinic location (urban or rural), distance from home to clinic, provider-patient relationship, and patient satisfaction with providers. The third group of variables was those related to social support: level of satisfaction with social support (by family, friends, peer educators, and social organizations), social connectedness, and perceptions of social isolation.

Substance use was categorized dichotomously. Heavy alcohol consumption was five or more 30-50-ml drinks of strong alcohol drinks or five 330-ml bottles of beer within a few hours at any time in the preceding month. Illicit drug intake occurring on one or more occasions during the preceding month was noted. The Center for Epidemiologic Studies’ Depression Scale (CES-D) [22,23] was used to measure depressive symptoms. Those with a score of 27 or higher were classified as having probable major depression [24]. Internal consistency (Cronbach’s α) for the CES-D was 0.89. Chance health locus of control was measured with a subscale of the multidimensional health locus of control (MHLC) scale [25] (Cronbach’s α = 0.78). The AACTG instrument was used to measure medication side-effect experiences [26]. A patient reaction assessment tool with three sub-scales on patients’ perception related to health care providers was used to measure the provider-patient relationship [27]. Cronbach’s α of perceived quality of the information from providers, affective behaviours, and perceived ability of providers to initiate communication were 0.85, 0.83 and 0.90, respectively. A four-point Likert scale was used to assess patients’ satisfaction with their social support from various groups (i.e., family members, friends, peer educators, and social organizations). Last, a scale by Hawthorne [28] was used to measure social connectedness (Cronbach’s α =0.70) and social isolation (Cronbach’s α = 0.77).

The outcome variables and most predictor variables were collected with the ACASI questionnaire. The following clinical variables were extracted from patient medical records: ART regimen, name and number of each ARV drug prescribed per dose, number of doses per day, duration on therapy, current TB treatment, and cotrimoxazole use.

### Data analysis

Data extracted from medical records were manually entered and merged with the ACASI data file into a single dataset. Microsoft Access (Microsoft Inc., Redmond, WA, USA) and MySQL database (MySQL Inc., Seattle, Washington, USA) were used for data entry and management. The Statistical Package for the Social Sciences 17.0 (SPSS Inc., Chicago, IL, USA) was used for statistical analysis.

Both measures of ART adherence (i.e., the VAS past-month dose adherence and the AACTG 4-day time adherence) were dichotomized into non-adherent and adherent. Agreement between the two measures was tested using the kappa (k) statistic. Univariate logistic regression analyses were conducted to assess the association of all hypothesized independent variables with the VAS past-month dose adherence. Multivariate logistic regression analysis was conducted by including all variables associated with suboptimal adherence at p ≤ 0.1 in the univariate analysis, along with variables that were hypothesized a priori to be strong predictors of ART adherence. Because significant co-linearity was found between heavy alcohol use and illicit drug use in the prior month, those variables were entered in combination in the multivariate analysis. All hypotheses were tested at the 95% probability level.

## Results

### Characteristics of study participants

Among 953 eligible patients, 759 were contacted in person, among whom 622 provided informed consent. Data from seven patients were excluded because of incomplete questionnaire response, resulting in 615 patients in the final sample. The characteristics of participants are summarized in Table 1. One third of participants were female and the ages of participants ranged from 18–60 years. More than half of the participants reported that they had consumed alcohol in the previous month, and of those 68.9% admitted to heavy drinking (more than five drinks in 2 hours) at least once during the month. About half had ever used illicit drugs, while 11.9% reported using an illicit drug in the prior month. When scores on the Depression scale were categorised according to the classification of Zich et al. [24], more than half of the patients had at least mild depressive symptoms (score of 16–26) and almost one fourth had major depressive symptoms (score of 27+).

**Table 1:** Characteristics of participants (N=615)

**Socio-demographic and behavioral characteristics**	**n**	**%**	** Clinical characteristics**	**n**	**%**
**Sex** (Female)	209	34.0%	** ART regimen **^**1**^		
**Age** (Mean: 32.7± 6.2)			d4T + 3TC + NVP	356	57.9%
18–30	250	40.7%	d4T + 3TC + EFV	165	26.8%
31–40	301	48.9%	AZT + 3TC + NVP	52	8.5%
>=41	64	10.4%	AZT + 3TC + EFV	17	2.8%
**Education**			Others	25	4.1%
<=Primary	57	9.3%	** Duration of ART (months)** (Mean: 20.2±13.2)		
Secondary	301	39.7%	<=12 months	201	32.7%
High school	199	32.4%	13–24 months	221	35.9%
College or University	115	18.7%	>24 month	193	31.4%
**Occupation**			** Tuberculosis treatment**		
Unemployed	119	19.3%	Ongoing	42	6.8%
Salary-paid job	284	46.2%	In the past	115	18.7%
Others (farmer, labor works…)	212	34.5%	Never	458	74.5%
**Marital status**			** Cotrimoxazole prophylaxis use**		
Single	114	18.5%	Ongoing	195	31.7%
Married or live w partners	354	57.6%	In the past	175	28.5%
Divorced/separated/widow	147	23.9%	Never	245	39.8%
**Alcohol use (last month)**	321	52.2%	** Facility location**		
**Had >5 drinks (last month)**	215	34.0%	Rural	409	66.5
**Illicit drug use (ever)**	319	51.9%	Urban	206	33.5
**Illicit drug use (last month)**	87	11.9%	** Administrative level of facility**		
**Use of adherence aid**	491	79.8%	Provincial/City level	353	57.4
**Depression**			District level	262	42.6
No depressive symptoms	135	22.0%	** Distance from residence to clinic**		
Mild depressive symptoms	334	54.3%	<=10 km	302	49.1
Major depressive symptoms	146	23.7%	>10 km	313	50.9

^*1*^*d4T, stavudine; 3TC, lamivudin; NVP, nevirapine; EFV, efavirenz, AZT, zidovudine.*

### Suboptimal ART adherence

The prevalence of one-month suboptimal adherence measured by VAS was 24.9% (153/615), while the prevalence of 4-day not-on-time-adherence using the AACTG instrument was 29% (179/615). The observed agreement of the two measurements was 84%, with a kappa coefficient of 0.596 (standard error = 0.04, p < 0.001). This indicates that the majority of individuals were consistently classified either as adherent or as non-adherent by the two measures, while 16% had discordant classification (Table 2).

**Table 2:** Agreement between two measures of ART adherence (N=615)

		**AACTG score**^***1***^		
		**Suboptimal adherence**	**Optimal adherence**	**Total**
**VAS score**^***2***^	Suboptimal adherence	117(19.0%)	36(5.9%)	153(24.9%)
	Optimal adherence	62(10.1%)	400(65.0%)	462(75.1%)
	Total	179(29.1%)	436(70. 9%)	615(100%)
	Suboptimal adherence	117(19.0%)	36(5.9%)	153(24.9%)
Optimal adherence	62(10.1%)	400(65.0%)	462(75.1%)	
Total	179(29.1%)	436(70. 9%)	615(100%)	

^*1*^ AACTG, Adult AIDS Clinical Trial Group; ^*2*^ VAS, Visual Analogue Scale.

### Factors associated with suboptimal ART adherence

Table 3 and 4 summarise the results of logistic regression analyses of factors associated with suboptimal ART adherence measured by VAS, which is more conservative than the AACTG results. Significant associations were observed in the univariate analyses for heavy alcohol use in the prior month, illicit drug use in the prior month, shorter distance from residence to clinic (<10 km), depressive symptoms, a greater number of medication side effects, a high level of chance health locus of control, a low perceived quality of information from health care providers, low satisfaction with received support, and low social connectedness. Socio-demographic characteristics (i.e. gender, age, and education) were not associated with suboptimal adherence to ART.

**Table 3:** Factors associated with suboptimal ART adherence measured by the visual analogue scale (N=615)

**Factors**		**% of optimal adherent group (n=462)**	**% of suboptimal adherent group (n=153)**	**Unadjusted OR**^***1 ***^**(95% CI)**	**Adjusted OR**^***2 ***^**(95% CI)**	**P value**^***3***^
**Socio-demographic factors**						
Sex	Male	74.0	26.0	1		
	Female	78.0	22.0	0.79(0.53–1.20)	–	–
Age	18–30	74.8	25.2	1	–	–
	31–40	74.8	25.2	1.00(0.68–1.48)		
	>= 40	78.1	21.9	0.83(0.43–1.61)		
Education	<= Primary	59.6	40.4	1		
	Higher	72.0	28.0	0.58(0.33–1.03)	–	–
**Clinical factors**						
Site	Kinh Mon & Chi Linh	67.9	32.1	1	1	
	Dong Da	72.9	27.1	0.79(0.41–1.49)	1.20(0.57–2.52)	0.628
	Dong Anh	82.0	18.0	0.46(0.24–0.90)	0.59(0.27–1.29)	0.188
	Hai Duong	71.3	28.7	0.85(0.44–1.65)	1.34(0.61–2.93)	0.464
Distance from residence to clinic	>10 km	78.6	21.4	0.68(0.47–0.99)	0.83(0.53–1.28)	0.396–
**Alcohol and drug use**						
Heavy alcohol use (last month)	No	81.6	18.4	1	–	–
	Yes	69.2	30.8	1.98(1.36–2.89)		
Illicit drug Use (last month)	No	77.7	22.3	1	–	–
	Yes	56.2	43.8	2.72(1.64–4.50)		
Interaction between drug and alcohol use	No drug + No alcohol	81.2	18.8	1	1	
	No drug + Alcohol	69.5	30.5	1.90(1.25–2.89)	2.14(1.34–3.41)	.001
	Drug + No alcohol	71.4	28.6	1.73(0.73–4.09)	1.49(0.59–3.80)	.402
	Drug + Alcohol	46.7	53.3	4.94(2.61–9.37)	5.04(2.46–10.32)	<0.001
**Depression**	No/mild symptom	78.7	21.3	1	1	
	Major symptom	63.7	36.3	2.10(1.40–3.14)	3.26(2.04–5.22)	<0.001

^1^ unadjusted odds ratio of suboptimal adherence is obtained from the univariate analysis; ^2^ adjusted odds ratio is obtained from multivariate analysis (95% confidence interval); ^3^ p values of the adjusted odds ratio.

**Table 4:** Continuous factors

**Factors**	**Mean of optimal adherent group (n=462)**	**Mean of suboptimal adherent group (n=153)**	**Unadjusted OR**^***1 ***^**(95% CI)**	**Adjusted OR**^***2 ***^**(95% CI)**	**P value**^***3***^
**Side-effects experienced**	20.64	27.19	1.02(1.01–1.04)	1.03(1.01–1.04)	<0.001
**Chance health locus of control**	16.6	18.69	1.06(1.02–1.09)	1.06(1.02–1.09)	0.001
**Social related factors**					
Perceived quality of information from HCPs	23.32	22.16	0.91(0.86–0.95)	0.93(0.88–0.99)	0.021
Satisfaction with received support	8.49	7.73	0.88(0.82–0.95)	0.94(0.86–1.02)	0.138
Social connectedness	10.89	9.65	0.90(0.86–0.95)	0.94(0.88–0.99)	0.033

^1^ unadjusted odds ratio of suboptimal adherence is obtained from the univariate analysis; ^2^ adjusted odds ratio is obtained from multivariate analysis (95% confidence interval); ^3^ p values of the adjusted odds ratio.

In multivariate analysis, factors that remained significantly associated with suboptimal adherence to ART were depression, heavy alcohol use in the prior month, medication side-effect experiences, chance health locus of control, low perceived quality of information from care providers, and low level of social connectedness. Although illicit drug use in the prior month without heavy alcohol use was not associated with suboptimal adherence, a significant interaction was found between illicit drug use and heavy alcohol use in the prior month. Participants who reported only heavy alcohol use had an adjusted odd ratio (aOR) of 2.14 (CI: 1.34-3.41), while those who reported both heavy alcohol use and illicit drug use had an aOR of 5.04 (CI: 2.46-10.32).

## Discussion

This study is based on a large and diverse sample of subjects enrolled in ART programs in Viet Nam, and indicates that suboptimal ART adherence is a critical issue that requires attention. Using the VAS measurement, we found that one in four (24.9%) people taking ART were not optimally adherent over the preceding month, and a higher proportion (29.1%) were classified as non-adherent with the AACTG measure of 4-day on-time medication use. These two prevalence estimates are higher than both prior studies in Viet Nam [4,5]. Compared with other published studies from East and South Asia, the suboptimal adherence we report is at the high end of the range (from 0.4% to 26.0%).

Direct comparison of suboptimal ART adherence prevalence from various studies is hindered by differences in study design and adherence measures, including time reference periods. Face-to-face interviews may be unreliable because patients may not be honest about their suboptimal adherence when they are interviewed by clinic staff about this sensitive issue. To our knowledge, the present study is the first to use the ACASI technique to investigate ART adherence in a developing country. This approach is widely used in other contexts to reduce social desirability response bias and enhance the veracity of self-report [6,9]. In Viet Nam, this technique has been useful to enhance disclosure of sensitive information in analyses with adolescents and injection drug users [9]. The improved privacy and confidentiality of the ACASI method may explain the relatively high estimates of suboptimal adherence we report here.

This study extends previous analyses of the determinants of ART suboptimal adherence that have been conducted in Asia and other regions in the world, and has clear implications for care for PLHIV in low resource environments. Among the many variables examined, depressive symptoms had one of the strongest associations with suboptimal adherence. This is consistent with research from high-income countries and in Africa [29-33] and strengthens the argument for mental health services that may improve ART adherence outcomes [33-36]. Currently, mental health services are not available at most HIV outpatient clinics in Viet Nam [4].

This study also confirmed that heavy alcohol use is a significant predictor of suboptimal ART adherence. Globally, alcohol use has been recognized as a major barrier for medication adherence [37]. A meta-analysis reported that among PLHIV, those who drink alcohol were approximately 40–50% less likely to be adherent compared with those who abstain or drink relatively little, and the effect size was even greater for problem drinkers [37]. The present study found that recent illicit drug use was not itself associated with suboptimal ART adherence, but we identified a significant interaction with recent heavy alcohol use. Compared with subjects with recent heavy alcohol use alone, ART adherence was much poorer among subjects who reported heavy alcohol use and illicit drug use. A number of interventions currently exist in Viet Nam to support drug users, including methadone maintenance therapy, needle and syringe exchange, condom distribution, and peer support groups. However, little effort has been directed to support alcohol abusers, including alcohol abusers living with HIV/AIDS. An important implication of our findings is that care and support for people who use alcohol and/or illicit drugs should be enhanced and sustained in Viet Nam, as a core part of comprehensive efforts to improve HIV treatment adherence. These efforts should be conducted in parallel with research, because few systematic evaluations have assessed the effectiveness of programs designed to optimize ART adherence for substance users, especially in resource-limited settings [38].

Another psychological factor identified was the fatalistic belief that chance or luck controls one’s destiny, including health outcomes. The multivariate results indicated that after adjusting for other variables, chance health locus of control remained a significant predictor of suboptimal ART adherence. This finding may be used to guide HIV clinic counsellors to discuss fatalistic beliefs as a barrier to self-care and healthy behaviours. Consistent with other research of the influence of patient-provider relationships on ART adherence [39,40], this study found that the perceived quality of information from health care providers was positively associated with adherence.

An interesting insight emerged from the analysis of how family and social support influence adherence. With a relatively large sample size, this study had enough statistical power to examine the relative importance of different types of social and family support in a multivariable model. We did not find significant relationships between ART adherence and different types of support from family, peers and social organisations. Both patient overall satisfaction with support received, and feelings of social connectedness, significantly correlated with adherence. In developing countries, a sense of social responsibility may influence patient adherence [41]. Specific interventions to enhance a sense of connectedness that maintains and strengthens social responsibility among patients, their families, and peers may help to optimize ART adherence.

The present study has some limitations. Selection bias may have occurred, because only patients on ART treatment who came to a clinic at the time of data collection and provided informed consent were included. This excluded patients who were not retained in HIV care, patients who missed clinical appointments during the study period, or patients who refused to participate in the study. Although the number of patients studied per clinic was proportional to the number of patients registered and receiving ART in each site, and researchers made considerable efforts to revisit these clinics several times to recruit patients, it is conceivable the non-participants were at higher risk of poor adherence. Therefore, the current study may underestimate the true level of suboptimal ART adherence. A further limitation is the cross-sectional nature of this study, which limits the ability of the analysis to determine the direction of causation.

## Conclusions

We report here the largest study of ART adherence among PLHIV in South East Asia. The participants were a diverse group from five clinics located in both rural and urban areas. The level of suboptimal adherence to ART appears to be higher than estimated in previous Asian studies. This may be due to the interview mode of the current study (ACASI) which enhances privacy and confidentiality and may improve the validity of self-reported information. Suboptimal adherence was strongly influenced by depression, ongoing substance use, medication side effects, and poor social connectedness. The study findings can be used to optimize ARV treatment adherence in Viet Nam.

## Abbreviations

AACTG: Adult AIDS clinical trials group; ACASI: Audio computer-assisted self-interview; AIDS: Acquired immune deficiency syndrome; ART: Antiretroviral therapy; CES-D: Center for epidemiological studies depression; HIV: Human immunodeficiency virus; MHLC: Multidimensional health locus of control; MMT: Methadone maintenance therapy; OR: Odd ratio; PLHIV: People living with HIV/AIDS; SD: Standard deviations; SE: Standard error; SPSS: Statistical package for the social sciences; VAS: Visual analogue scale; WHO: World health organization

## Competing interests

The authors declare that they have no competing interests.

## Authors’ contributions

HD was involved in the study design, data collection, data analysis and interpretation, and drafted the manuscript. MD participated in the study conceptualization, design, supervision, analysis and writing. MK participated in the study design, coordination and helped to draft the manuscript. CP participated in the study design, guided the statistical analysis and reviewed drafts of the manuscript. KN was involved in the study design, facilitated clinical support, contributed to data interpretation and revised the manuscript. All authors read and approved the final manuscript.

## Authors’ information

HD is chair of the Health System Management Department of the Hanoi School of Public Health in Vietnam (HSPH). She has 16 years of professional experience in teaching and doing research in the areas of HIV/AIDS. MD is a Professor of Social Epidemiology at Queensland University of Technology (QUT) and Director of the QUT-Vietnam Public Health Program. He has extensive experience in health social science and HIV/AIDS. MK is senior medical officer in HIV care and treatment of the WHO Viet Nam Country Office. CP is chair of the Public Health Informatics Department of the HSPH. KN is the director of the National Institute for Infectious and Tropical Diseases (NIITD) and a senior expert on HIV/AIDS and infectious diseases. All authors read and approved the final manuscript.

## Pre-publication history

The pre-publication history for this paper can be accessed here:

http://www.biomedcentral.com/1471-2334/13/154/prepub
